# Substrate specificity of new methyl-directed DNA endonuclease GlaI

**DOI:** 10.1186/1471-2199-9-7

**Published:** 2008-01-15

**Authors:** Galina V Tarasova, Tatiana N Nayakshina, Sergey KH Degtyarev

**Affiliations:** 1 SibEnzyme Ltd., Novosibirsk, Russian Federation

## Abstract

**Background:**

Recently, we have discovered site-specific endonucleases, which recognize and cleave only DNA sequences with 5-methylcytosine. Two specificities of such endonucleases have been described. Enzymes BisI, BlsI, and GluI are isoschizomers and hydrolyze the DNA sequence 5'-GCNGC-3'/3'-CGNCG-5', which is methylated in different ways. The enzyme GlaI cleaves the DNA sequence 5'-GCGC-3'/3'-CGCG-5' if there are two, three or four 5-methylcytosines. The goal of the present work is to study in detail the composition of recognition sequence and effect of the methylated cytosines on the efficiency of DNA cleavage by the methyl-directed DNA endonuclease GlaI

**Results:**

In a recent work we have studied the dependence of GlaI activity on the quantity and location of 5-methylcytosines in the enzyme recognition sequence 5'-GCGC-3'/3'-CGCG-5'. A significant DNA cleavage has been observed for oligonucleotide duplexes, which include either three or four 5-methylcytosines. In this work we have studied dependence of the GlaI activity on quantity and location of methylated cytosines, as well as on composition of the recognition sequence.

**Conclusion:**

The list of good substrates for GlaI includes a fully methylated site 5'-CGCG-3'/3'-GCGC-5', sites with three cytosines of a general structure 5'-PuMGM-3'/3'-PyGMG-5', and one recognition sequence with two methylated cytosines 5'-AMGT-3'/3'-TGMA-5', where M is 5-methylcytosine.

GlaI intermediate substrates include sites with three methylated cytosines of a general structure 5'-GMPuM-3'/3'-MGPyG-5', as well as a site with two methylcytosines 5'-GMGT-3'/3'-CGMA-5'.

The site 5'-GMGC-3'/3'-CGMG-5' may be considered a low activity substrate.

## Background

Most bacterial site-specific DNA endonucleases are restriction endonucleases. Restriction endonucleases (restriction enzymes) and DNA-methyltransferases (methylases) of the same specificity form the so-called restriction-modification (R-M) system. It is supposed that restriction endonucleases (restriction enzymes) protect the cell against penetration of foreign DNA (for example phage DNA), whereas methylases protect the host DNA against cleavage from their own restriction enzyme. Methylases protect DNA by modifying the recognition sequence. Even the name "restriction endonuclease" originated from experimentally observed phenomenon – restriction of bacterial phage propagation in microorganisms [1]. Recently we have discovered site-specific endonucleases, which exist in bacterial cells without corresponding methylases and hydrolyze only cytosine-methylated DNA [2-5]. Based on the current classification [6,7] these enzymes may be placed into IIM group of site-specific endonucleases, i.e. endonucleases recognizing only methylated DNA. Three specificities of endonucleases which belong to the IIM group have been described. The enzyme DpnI and its isoshizomers recognize the DNA sequence 5'-G(m6A)TC-3' in which adenine is methylated [8]. Enzymes BisI [2], BlsI [4], and GluI [5] are isoschizomers and hydrolyze the DNA sequence 5'-GCNGC-3', which is fully or partially methylated in C5 base position. The enzyme GlaI recognizes the 5mC-modified sequence 5'-GCGC-3' [3]. All other known 5mC-specific restriction enzymes, including McrBC, do not have well defined recognition sequences and cleavage sites [6]. Thus, GlaI and another 5mC-specific endonuclease BisI are new prototypes of site specific DNA endonucleases and their properties need to be studied in details. Recently we have shown that the DNA-endonuclease GlaI hydrolyses DNA sequence 5'-GCGC-3' with varying efficiency, depending on quantities and positions of C5 methylated cytosines but does not cleave C4 methylated cytosine sequences [9]. The goal of the present work is to study the effect of the cytosine methylation and nucleotide sequence of the recognition site on the efficiency of GlaI DNA cleavage.

## Methods

Restriction endonucleases, T4 polynucleotide kinase and buffer solutions used in this study were provided by SibEnzyme Ltd. (Russia). Oligodeoxyribonucleotide substrates for endonuclease GlaI were synthesized at SibEnzyme Ltd. (Russia). Compositions of the oligodeoxyribonucleotides are provided in Table 1.

**Table 1:** Structure of oligodeoxyribonucleotides

Name of oligonucleotide	Structure of oligonucleotide
G1	5'-CTATGAACGTTTTC**GCGC**TGACGGACCGTATC-3'
G2	5'-GATACGGTCCGTCA**GCGC**GAAAACGTTCATAG-3'
G3	5'-CTATGAACGTTTTC**GMGC**TGACGGACCGTATC-3'
G4	5'-GATACGGTCCGTCA**GMGC**GAAAACGTTCATAG-3'
G5	5'-CTATGAACGTTTTC**GCGM**TGACGGACCGTATC-3'
G6	5'-GATACGGTCCGTCA**GCGM**GAAAACGTTCATAG-3'
G7	5'-CTATGAACGTTTTC**GMGM**TGACGGACCGTATC-3'
G8	5'-GATACGGTCCGTCA**GMGM**GAAAACGTTCATAG-3'
G11	5'-CTATGAACGTTTTC**AMGM**TGACGGACCGTATC-3'
G12	5'-GATACGGTCCGTCA**GMGT**GAAAACGTTCATAG-3'
G13	5'-CTATGAACGTTTTC**GTGM**TGACGGACCGTATC-3'
G14	5'-GATACGGTCCGTCA**GMAM**GAAAACGTTCATAG-3'
G15	5'-CTATGAACGTTTTC**AMGT**TGACGGACCGTATC-3'
G16	5'-GATACGGTCCGTCA**AMGT**GAAAACGTTCATAG-3'
G17	5'-CTATGAACGTTTTC**AMGC**TGACGGACCGTATC-3'
G18	5'-CTATGAACGTTTTC**GMGT**TGACGGACCGTATC-3'
G19	5'-GATACGGTCCGTCA**AMGC**GAAAACGTTCATAG-3'
G20	5'-CTATGAACGTTTTC**GMAM**TGACGGACCGTATC-3'
G21	5'-GATACGGTCCGTCA**GTGM**GAAAACGTTCATAG-3'
G22	5'-CTATGAACGTTTTC**MMGM**TGACGGACCGTATC-3'
G23	5'-GATACGGTCCGTCA**GMGG**GAAAACGTTCATAG-3'
G24	5'-CTATGAACGTTTTC**GGGM**TGACGGACCGTATC-3'
G25	5'-GATACGGTCCGTCA**GMMM**GAAAACGTTCATAG-3'
G26	5'-CTATGAACGTTTTC**CMGM**TGACGGACCGTATC-3'
G27	5'-GATACGGTCCGTCA**GMCM**GAAAACGTTCATAG-3'
G28	5'-CTATGAACGTTTTC**TMGM**TGACGGACCGTATC-3'
G29	5'-GATACGGTCCGTCA**GMGA**GAAAACGTTCATAG-3'
G30	5'-CTATGAACGTTTTC**GAGM**TGACGGACCGTATC-3'
G31	5'-GATACGGTCCGTCA**GMTM**GAAAACGTTCATAG-3'
G32	5'-CTATGAACGTTTTC**ACGM**TGACGGACCGTATC-3'
G33	5'-CTATGAACGTTTTC**ATGM**TGACGGACCGTATC-3'
G34	5'-GATACGGTCCGTCA**GMAT**GAAAACGTTCATAG-3'
G35	5'-GATACGGTCCGTCA**GCAM**GAAAACGTTCATAG-3'
G36	5'-GATACGGTCCGTCA**GMAC**GAAAACGTTCATAG-3'
G37	5'-CTATGAACGTTTTC**TMGA**TGACGGACCGTATC-3'
G38	5'-GATACGGTCCGTCA**TMGA**GAAAACGTTCATAG-3'

All duplexes were prepared by annealing two complementary oligodeoxyribonucleotides which differ from each other in the presence or the absence of methylcytosine and composition of the nucleotides in the recognition sequence (underlined in the Table above).

### Preparation of a substrate for endonuclease GlaI

The upper strand of each DNA duplex was labeled at the 5'-end with T4 polynucleotide kinase and [^32^P]ATP. After purification with Microspin™ G-25 Columns (Amersham Biosciences UK Limited, England), oligonucleotide was mixed with complementary unlabeled oligonucleotide in 1:1.5 ratio. The duplex was then annealed by heating to 65°C, followed by slow cooling to room temperature.

### Hydrolysis of oligonucleotide duplexes with endonuclease GlaI

Hydrolysis reactions were initiated by adding GlaI to 10 μl of the reaction mixture containing GlaI buffer (Tris-HCl pH 8.5 (at 25°C), 5 mM MgCl_2_, 10 mM NaCl, 1.5 mM β-mercaptoethanol) and oligonucleotide duplex with concentration of 62.5 nM. Reactions were carried out at 30°C.

### Determination of the product quantities after oligonucleotide cleavage

Electrophoresis of the hydrolysis products was carried out in denaturing 20% PAAG (polyacrylamide gel) with 7 M urea in tris-borate buffer. Radiolabeled DNA was detected and quantified by using Personal Molecular Imager (Bio-Rad laboratories, Inc., USA). For each labeled product, we determined DLU (Digital Light Units) proportional to the intensity of [^32^P] isotope radiation minus the background. Hydrolysis percentage was determined by dividing the reaction product DLU by the total DLU, calculated as the sum of the product DLU and the remaining unhydrolyzed duplex DLU. Data treatment was performed using the program Quantity One – 4.6.2. (Bio-Rad laboratories, Inc., USA).

### Determination of the enzyme activity

The minimal quantity of the enzyme required to complete hydrolysis of the oligonucleotide duplex G7*/G8 in GlaI buffer in 1 hour at the optimal temperature of 30°C was defined as one unit of enzyme activity.

GlaI activity (in %) for any substrate was determined as a ratio of tangent in linear range of activities curves for the studied substrate and an optimal substrate G7*/G8.

### Statistical treatment of results

The treatment was performed using the method of a standard deviation as the statistical test and program Origin 7.0. Microsoft Office Excel 2003 was used for graphical representation of the results.

## Results and discussion

Recently, we have discovered a new site-specific endonuclease GlaI, which does not cleave standard DNA substrates (lambda and T7 DNAs, adenoviral DNA, plasmids pUC19, pBR322) and which hydrolyzes only the plasmid DNA containing a gene of DNA-methyltransferase HspAI [3]. Enzyme M.HspAI methylates the internal cytosine at position 5 in the sequence 5'-GCGC-3' [3]. This fact allowed us to propose and then to confirm the hypothesis that the site-specific endonuclease GlaI cleaves the sequence 5'-GMGC-3'/3'-CGMG-5' [3]. In a recent work [9] we have studied the dependence of GlaI activity on the quantity and location of 5-methylcytosines in the enzyme recognition sequence. A significant DNA cleavage has been observed for oligonucleotide duplexes which contain either three or four 5-methylcytosines. A DNA duplex with four 5-methylcytosines (5'-GMGM-3'/3'-MGMG-5') displays a maximum level of cleavage efficiency and is considered to be a canonical substrate. The efficiency decreases when the quantity of methylated bases is lower. However, the influence on GlaI activity of the recognition sequence composition was not clear. In this work we have studied dependence of the GlaI activity on quantity and location of methylated cytosines, as well as on composition of the recognition sequence. Unlike with all other known restriction endonucleases, it is not possible to produce a whole set of DNA substrates for GlaI in the form of plasmid, phage or other native DNA, which have recognition sites methylated in different ways. In order to study the properties of endonuclease GlaI, we used chemically synthesized oligonucleotide duplexes, which include methylcytosine(s) and nucleotide substitutions in the recognition sequence 5'-GCGC-3'/3'-CGCG-5'.

**Figure 1:**
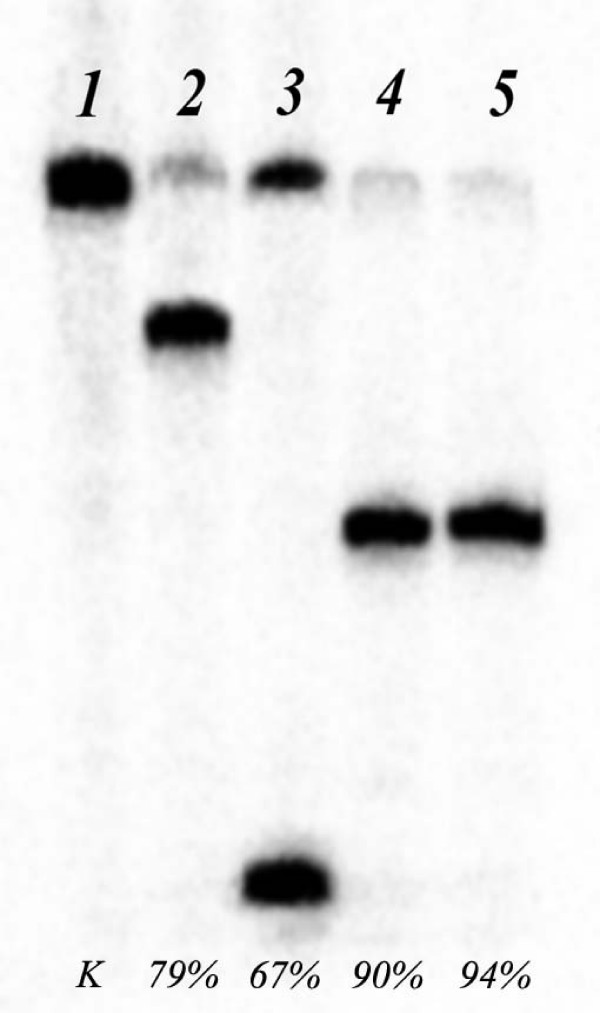
Cleavage of the oligonucleotide duplex G7*/G8 by endonucleases. Runs: 1 – DNA duplex without the enzyme; 2 – hydrolysis with restriction endonuclease Rsr2I; 3 – hydrolysis with restriction endonuclease AclI; 4, 5 – hydrolysis with endonuclease GlaI (30 units and 100 units, respectively).

Fig. 1 presents the results of the oligonucleotide duplex G7*/G8 (^32^P-labeled chain is marked by *) cleavage by different endonucleases. This duplex contains the completely methylated recognition sequence 5'-GMGM-3'/3'-MGMG-5'. Fig. 1 shows that duplex G7*/G8 is hydrolyzed by restriction endonucleases Rsr2I and AclI (recognition sites 5'-CGG^WCCG-3' and 5'-AAC^GTT-3' (respectively) as well as with enzyme GlaI.

As Figure 1 shows, duplex G8*/G7 is hydrolyzed with GlaI even more efficient than with restriction endonucleases Rsr2I and AclI.

In order to study dependence of GlaI activity on the quantity and location of modified cytosines in the recognition sequence, we analyzed the hydrolysis efficiency of various methylated oligonucleotides.

**Figure 2:**
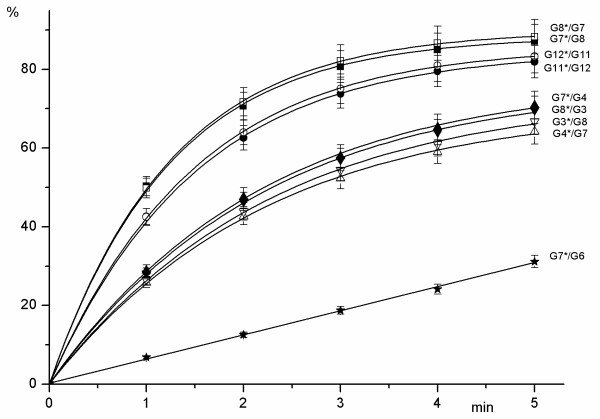
Dependence of the hydrolyzed product (in %) (axis Y) on the time of reaction: G7*/G8 (5'-TTTTC**GMGM**TGACG-3'/3'-AAAAG**MGMG**ACTGC-5') – ■, G8*/G7 (5'-CGTCA**GMGM**GAAAA-3'/3'-GCAGT**MGMG**CTTTT-5') – □, G3*/G8 (5'-TTTTC**GMGC**TGACG-3'/3'-AAAAG**MGMG**ACTGC-5') – ▽, G8*/G3 (5'-CGTCA**GMGM**GAAAA-3'/3'-GCAGT**CGMG**CTTTT-5') – ▼, G4*/G7 (5'-CGTCA**GMGC**GAAAA-3'/3'-GCAGT**MGMG**CTTTT-5') – △, G7*/G4 (5'-TTTTC**GMGM**TGACG-3'/3'-AAAAG**CGMG**ACTGC-5') – ▲, G11*/G12 (5'-TTTTC**AMGM**TGACG-3'/3'-AAAAG**TGMG**ACTGC-5') – ●, G12*/G11 (5'-CGTCA**GMGT**GAAAA-3'/3'-GCAGT**MGMA**CTTTT-5') – ○, G7*/G6 (5'-TTTTC**GMGM**TGACG-3'/3'-AAAAG**MGCG**ACTGC-5') – .

**Figure 3:**
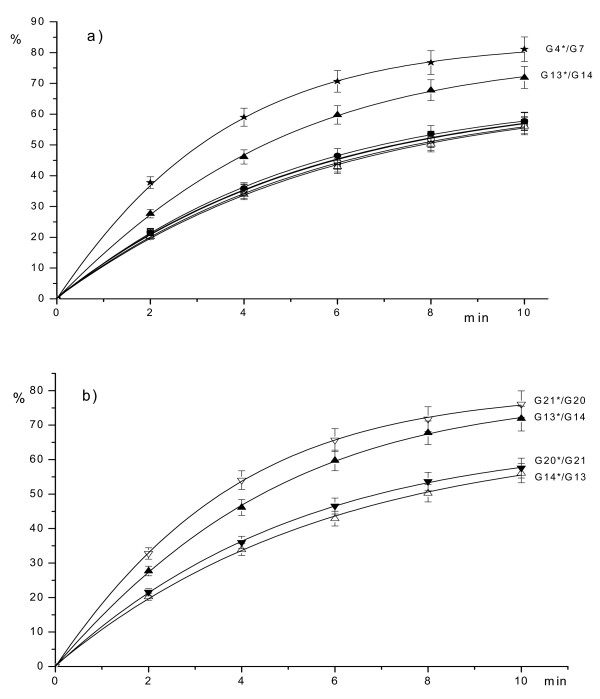
Dependence of the hydrolyzed product (in %) (axis Y) on the time of reaction: a) G7*/G6 (5'-TTTTC**GMGM**TGACG-3'/3'-AAAAG**MGCG**ACTGC-5') – ■, G6*/G7 (5'-CGTCA**GCGM**GAAAA-3'/3'-GCAGT**MGMG**CTTTT-5') – □, G5*/G8 (5'-TTTTC**GCGM**TGACG-3'/3'-AAAAG**MGMG**ACTGC-5') – ●, G8*/G5 (5'-CGTCA**GMGM**GAAAA-3'/3'-GCAGT**MGCG**CTTTT-5') – ○, G13*/G14 (5'-TTTTC**GTGM**TGACG-3'/3'-AAAAG**MAMG**ACTGC-5') – ▲, G14*/G13 (5'-CGTCA**GMAM**GAAAA-3'/3'-GCAGT**MGTG**CTTTT-5') – △, G4*/G7 (5'-CGTCA**GMGC**GAAAA-3'/3'-GCAGT**MGMG**CTTTT-5') – ; b) G13*/G14 (5'-TTTTC**GTGM**TGACG-3'/3'-AAAAG**MAMG**ACTGC-5') – ▲, G14*/G13 (5'-CGTCA**GMAM**GAAAA-3'/3'-GCAGT**MGTG**CTTTT-5') – △, G20*/G21 (5'-TTTTC**GMAM**TGACG-3'/3'-AAAAG**MGTG**ACTGC-5') – ▼, G21*/G20 (5'-CGTCA**GTGM**GAAAA-3'/3'-GCAGT**MAMG**CTTTT-5') – ▽.

**Figure 4:**
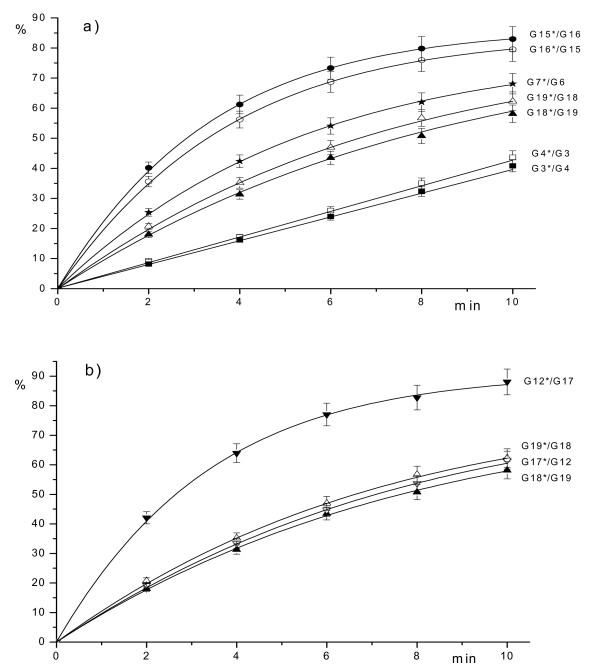
Dependence of the hydrolyzed product (in %) (axis Y) on the time of reaction: a) G3*/G4 (5'-TTTTC**GMGC**TGACG-3'/3'-AAAAG**CGMG**ACTGC-5') – ■, G4*/G3 (5'-CGTCA**GMGC**GAAAA-3'/3'-GCAGT**CGMG**CTTTT-5') – □, G15*/G16 (5'-TTTTC**AMGT**TGACG-3'/3'-AAAAG**TGMA**ACTGC-5') – ●, G16*/G15 (5'-CGTCA**AMGT**GAAAA-3'/3'-GCAGT**TGMA**CTTTT-5') – ○, G18*/G19 (5'-TTTTC**GMGT**TGACG-3'/3'-AAAAG**CGMA**ACTGC-5') – ▲, G19*/G18 (5'-CGTCA**AMGC**GAAAA-3'/3'-GCAGT**TGMG**CTTTT-5') – △, G7*/G6 (5'-TTTTC**GMGM**TGACG-3'/3'-AAAAG**MGCG**ACTGC-5') – ; b) G12*/G17 (5'-CGTCA**GMGT**GAAAA-3'/3'-GCAGT**CGMA**CTTTT-5') – ▼, G17*/G12 (5'-TTTTC**AMGC**TGACG-3'/3'-AAAAG**TGMG**ACTGC-5') – ▽, G18*/G19 (5'-TTTTC**GMGT**TGACG-3'/3'-AAAAG**CGMA**ACTGC-5') – ▲, G19*/G18 (5'-CGTCA**AMGC**GAAAA-3'/3'-GCAGT**TGMG**CTTTT-5') – △.

Experimental data have been processed as indicated in "Methods" and dependence of the hydrolyzed product (expressed in percent) on the time of the incubation are presented in Figures 2, 3, 4. The lines shown in these figures represent a typical Michaelis-Menten kinetics

As Figure 2 shows, the highest enzyme activity is observed for a fully methylated duplex G7*/G8 with four 5-methylcytosines. Recognition sequences with three methylated cytosines are hydrolyzed less effectively. There are two groups of these substrates: oligonucleotides with two internal 5-methylcytosines 5'-PuMGM-3'/3'PyGMG-5' and oligonucleotides with one internal 5-methylcytosine 5'-GMPuM-3'/3'-MGPyG. GlaI activity in hydrolysis of all other possible recognition sequences with three 5-methylcytosines is less than 1% of G7*/G8 cleavage rate. Fig. 2 shows that GlaI activity is about 60% for cleavage of both strands of duplex 5'-AMGM-3'/3'TGMG-5', 36%–41% for the upper strand and 27%–31% for a bottom strand of duplex 5'-GMGM-3'/3'CGMG-5'. Cleavage efficiency of the oligonucleotides with two external 5-methylcytosines is 16–17% for both strands, except for the strand containing 5'-GTGM-3' as this strand is cleaved with 21%–25% of the G7*/G8 efficiency (Fig. 3).

Some recognition sequences with two methylated cytosines are also substrates for GlaI. The general structure of these substrates is 5'-PuMGPy-3'/3'PyGMPu-5'. GlaI activity in hydrolysis of all other possible recognition sequences with two 5-methylcytosines is less than 1% of G7*/G8 cleavage rate. Fig. 4 shows that GlaI activity is 23–26% for cleavage of both strands of duplex 5'-AMGT-3'/3'-TGMA-5' and 13% for cleavage of both strand of duplex 5'-GMGT-3'/3'-CGMA-5'. There is an exception for a strand, which contains sequence 5'-GMGT-3', followed by purine nucleotides. This strand is hydrolyzed two times faster than the complementary chain. Finally, we have observed approximately 6% enzyme activity in hydrolysis of the sequence 5'-GMGC-3'/3'-CGMG-5' when compared to GlaI activity with G7*/G8 substrate.

The experimental results for all oligonucleotide duplexes are presented in Table 2. GlaI displays a maximal activity in hydrolysis of the fully methylated sequence 5'-GCGC-3'/3'-CGCG-5'. We determine the enzyme's activity in hydrolysis of other DNA duplexes as a percent of this oligonucleotide cleavage activity. The table shows that GlaI effectively cleaves DNA sequence 5'-GCGC-3'/3'-CGCG-5' containing 3 modified bases, when both internal cytosines are methylated. Sites with two external and one internal cytosine, as well as with two internal modified cytosines, are cleaved less efficiently. Oligonucleotide with two modified bases on one chain is cleaved insignificantly and only at larger concentrations of the enzyme. Oligonucleotide duplexes, which carry two methylcytosines in one chain (5'-GMGM-3'/3'-CGCG-5'), are cleaved by GlaI at enzyme concentrations more than 100 units per microlitre [9]. According to Table 2, GlaI activity less than 1% is observed for duplexes with two external 5-mehylcytosines (5'-GPyGM-3'/3'-MPuCG-5'), or one external and one internal 5-mehylcytosines in different chains (5'-PuPyGM-3'/3'-PyPuMG-5' and 5'-GMPuPy-3'/3'-MGPyPu-5'). We do not detect GlaI hydrolysis of oligonucleotides when purines are replaced by pyrimidines in the recognition sequence (5'-PyMGM-3'/3'-PuGMG-5', 5'-GMGPu-3'/3'-MGMPy-5', 5'-GPuGM-3'/3'-MPyMG-5', 5'-GMPyM-3'/3'-MGPuG-5' and 5'-PyMGPu-3'/3'-PuGMPy-5').

**Table 2:** GlaI activity on methylated oligonucleotide duplexes

Oligonucleotide duplex	The central part of structure of a duplex	Relative enzyme's activity (%) and standard error
G7*/G8	5'-TTTTC**GMGM**TGACG-3'	100 ± 5
	3'-AAAAG**MGMG**ACTGC-5'	
G8*/G7	5'-CGTCA**GMGM**GAAAA-3'	97 ± 5
	3'-GCAGT**MGMG**CTTTT-5'	
G3*/G8	5'-TTTTC**GMGC**TGACG-3'	28 ± 2
	3'-AAAAG**MGMG**ACTGC-5'	
G8*/G3	5'-CGTCA**GMGM**GAAAA-3'	40 ± 2
	3'-GCAGT**CGMG**CTTTT-5'	
G4*/G7	5'-CGTCA**GMGC**GAAAA-3'	31 ± 2
	3'-GCAGT**MGMG**CTTTT-5'	
G7*/G4	5'-TTTTC**GMGM**TGACG-3'	36 ± 2
	3'-AAAAG**CGMG**ACTGC-5'	
G11*/G12	5'-TTTTC**AMGM**TGACG-3'	61 ± 3
	3'-AAAAG**TGMG**ACTGC-5'	
G12*/G11	5'-CGTCA**GMGT**GAAAA-3'	60 ± 3
	3'-GCAGT**MGMA**CTTTT-5'	
G6*/G7	5'-CGTCA**GCGM**GAAAA-3'	16 ± 1
	3'-GCAGT**MGMG**CTTTT-5'	
G7*/G6	5'-TTTTC**GMGM**TGACG-3'	17 ± 1
	3'-AAAAG**MGCG**ACTGC-5'	
G5*/G8	5'-TTTTC**GCGM**TGACG-3'	17 ± 1
	3'-AAAAG**MGMG**ACTGC-5'	
G8*/G5	5'-CGTCA**GMGM**GAAAA-3'	17 ± 1
	3'-GCAGT**MGCG**CTTTT-5'	
G13*/G14	5'-TTTTC**GTGM**TGACG-3'	21 ± 1
	3'-AAAAG**MAMG**ACTGC-5'	
G14*/G13	5'-CGTCA**GMAM**GAAAA-3'	16 ± 1
	3'-GCAGT**MGTG**CTTTT-5'	
G20*/G21	5'-TTTTC**GMAM**TGACG-3'	17 ± 1
	3'-AAAAG**MGTG**ACTGC-5'	
G21*/G20	5'-CGTCA**GTGM**GAAAA-3'	25 ± 1
	3'-GCAGT**MAMG**CTTTT-5'	
G3*/G4	5'-TTTTC**GMGC**TGACG-3'	5 ± 1
	3'-AAAAG**CGMG**ACTGC-5'	
G4*/G3	5'-CGTCA**GMGC**GAAAA-3'	6 ± 1
	3'-GCAGT**CGMG**CTTTT-5'	
G12*/G17	5'-CGTCA**GMGT**GAAAA-3'	28 ± 1
	3'-GCAGT**CGMA**CTTTT-5'	
G17*/G12	5'-TTTTC**AMGC**TGACG-3'	13 ± 1
	3'-AAAAG**TGMG**ACTGC-5'	
G15*/G16	5'-TTTTC**AMGT**TGACG-3'	26 ± 1
	3'-AAAAG**TGMA**ACTGC-5'	
G16*/G15	5'-CGTCA**AMGT**GAAAA-3'	23 ± 1
	3'-GCAGT**TGMA**CTTTT-5'	
G18*/G19	5'-TTTTC**GMGT**TGACG-3'	12 ± 1
	3'-AAAAG**CGMA**ACTGC-5'	
G19*/G18	5'-CGTCA**AMGC**GAAAA-3'	14 ± 1
	3'-GCAGT**TGMG**CTTTT-5'	
G7*/G2	5'-TTTTC**GMGM**TGACG-3'	< 1
	3'-AAAAG**CGCG**ACTGC-5'	
G2*/G7	5'-CGTCA**GCGC**GAAAA-3'	< 1
	3'-GCAGT**MGMG**CTTTT-5'	
G13*/G35	5'-TTTTC**GTGM**TGACG-3'	< 1
	3'-AAAAG**MACG**ACTGC-5'	
G35*/G13	5'-CGTCA**GCAM**GAAAA-3'	< 1
	3'-GCAGT**MGTG**CTTTT-5'	
G5*/G6	5'-TTTTC**GCGM**TGACG-3'	< 1
	3'-AAAAG**MGCG**ACTGC-5'	
G6*/G5	5'-CGTCA**GCGM**GAAAA-3'	< 1
	3'-GCAGT**MGCG**CTTTT-5'	
G12*/G32	5'-CGTCA**GMGT**GAAAA-3'	< 1
	3'-GCAGT**MGCA**CTTTT-5'	
G32*/G12	5'-TTTTC**ACGM**TGACG-3'	< 1
	3'-AAAAG**TGMG**ACTGC-5'	
G33*/G34	5'-TTTTC**ATGM**TGACG-3'	< 1
	3'-AAAAG**TAMG**ACTGC-5'	
G34*/G3	5'-CGTCA**GMAT**GAAAA-3'	< 1
	3'-GCAGT**MGTA**CTTTT-5'	
G13*/36	5'-TTTTC**GTGM**TGACG-3'	< 1
	3'-AAAAG**CAMG**ACTGC-5'	
G36*/13	5'-CGTCA**GMAC**GAAAA-3'	< 1
	3'-GCAGT**MGTG**CTTTT-5'	
G3*/G6	5'-TTTTC**GMGC**TGACG-3'	< 1
	3'-AAAAG**MGCG**ACTGC-5'	
G6*/G3	5'-CGTCA**GCGM**GAAAA-3'	< 1
	3'-GCAGT**CGMG**CTTTT-5'	
G4*/G5	5'-CGTCA**GMGC**GAAAA-3'	< 1
	3'-GCAGT**MGCG**CTTTT-5'	
G5*/G4	5'-TTTTC**GCGM**TGACG-3'	< 1
	3'-AAAAG**CGMG**ACTGC-5'	
G22*/G23	5'-TTTTC**MMGM**TGACG-3'	< 1
	3'-AAAAG**GGMG**ACTGC-5'	
G23*/G22	5'-CGTCA**GMGG**GAAAA-3'	< 1
	3'-GCAGT**MGMM**CTTTT-5'	
G24*/G25	5'-TTTTC**GGGM**TGACG-3'	< 1
	3'-AAAAG**MMMG**ACTGC-5'	
G25*/G24	5'-CGTCA**GMMM**GAAAA-3'	< 1
	3'-GCAGT**MGGG**CTTTT-5'	
G28*/G29	5'-TTTTC**TMGM**TGACG-3'	< 1
	3'-AAAAG**AGMG**ACTGC-5'	
G29*/G28	5'-CGTCA**GMGA**GAAAA-3'	< 1
	3'-GCAGT**MGMT**CTTTT-5'	
G30*/G31	5'-TTTTC**GAGM**TGACG-3'	< 1
	3'-AAAAG**MTMG**ACTGC-5'	
G31*/G30	5'-CGTCA**GMTM**GAAAA-3'	< 1
	3'-GCAGT**MGAG**CTTTT-5'	
G37*/G38	5'-TTTTC**TMGA**TGACG-3'	< 1
	3'-AAAAG**AGMT**ACTGC-5'	
G38*/G37	5'-CGTCA**TMGA**GAAAA-3'	< 1
	3'-GCAGT**AGMT**CTTTT-5'	
G26*/G23	5'-TTTTC**CMGM**TGACG-3'	< 1
	3'-AAAAG**GGMG**ACTGC-5'	
G23*/G26	5'-CGTCA**GMGG**GAAAA-3'	< 1
	3'-GCAGT**MGMC**CTTTT-5'	
G27*/G24	5'-CGTCA**GMCM**GAAAA-3'	< 1
	3'-GCAGT**MGGG**CTTTT-5'	
G24*/G27	5'-TTTTC**GGGM**TGACG-3'	< 1
	3'-AAAAG**MCMG**ACTGC-5'	

**Figure 5:**
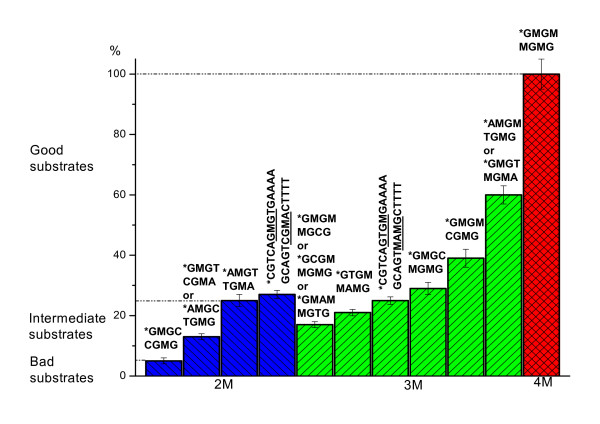
GlaI activity on methylated oligonucleotide duplexes.

In Fig. 5 we have summarized the data obtained in this study. Tested substrates have been divided into several groups (blue, green and red colour). GlaI cleaves DNA substrates with the following recognition sequences:

a. 5'-GMGM-3'/3'-MGMG-5' (four 5-methylcytosines, red colour);

b. 5'-PuMGM-3'/3'PyGMG-5' (three 5-methylcytosines, green colour);

c. 5'-GMPuM-3'/3'-MGPyG-5' (three 5-methylcytosines, green colour);

d. 5'-PuMGPy-3'/3'PyGMPu-5' (two 5-methylcytosines, blue colour).

A fully methylated sequence 5'-GCGC-3'/3'-CGCG-5' is the best substrate, which we consider as a canonical one. The above listed substrates with three methylcytosines are divided into two subgroups (b and c). The first subgroup includes good substrates with two internal cytosines of general structure 5'-PuMGM-3'/3'PyGMG-5' and the second subgroup includes intermediate substrates with two external 5-methylcytosines of general structure 5'-GMPuM-3'/3'-MGPyG-5'. Cleavage activity of GlaI in the first subgroup varies from 25% up to 66% of that for the site with four 5-methylcytosines (a), whereas the second subgroup cleavage activity is between 10% and 20%. Variation of GlaI activity in cleavage of recognition sequences with two internal 5-methylcytosines (subgroup d) is 5% – 26%.

In our analysis of data in Figure 5, we should note the following peculiarities of GlaI activity.

A. For some sites there is a difference in cleavage efficiency of two strands of recognition sequence. This is occurs for site 5'-GMGM-3'/3'-CGMG-5', but not for 5'-AMGM-3'/3'-TGMG-5'; and also occurs for site 5'-GTGM-3'/3'-MAMG-5'. There is no significant difference in GlaI cleavage activity of two strands for other sites.

B. We observe that the nucleotides which surround the 4 base recognition sequence can influence the enzyme's cleavage activity for sites 5'-GTGM-3'/3'-MAMG-5' and 5'-GMGT-3'/3'-CGMA-5'. GlaI displays elevated levels of activity, about 26%, in the upper strand cleavage of those recognition sequences which are given in bold in Figure 5. The upper strand's recognition sequence is either 5'-GMGT-3' or 5'-GTGM-3', followed by a block of purine nucleotides. It can be concluded that GlaI displays higher activity in cleavage of one chain of the recognition sequence if this strand contains 5-methylcytosine, T and two G bases, followed by A and G sequence.

C. For DNA cleavage, nucleotide A is a better base than G in the purine position of recognition sequences 5'-PuMGM-3'/3'PyGMG-5' and 5'-PuMGPy-3'/3'PyGMPu-5'. There is no difference in activity for either nucleotide A or G in the site 5'-GMPuM-3'/3'-MGPyG-5'. The greatest activity variance, of more than 4-fold, occurs in the cleavage of oligonucleotides with two internal 5-methylcytosines. Surprisingly, one of them, 5'-AMGT-3'/3'-TGMA-5', is a good substrate for cleavage with enzyme GlaI (about 25% activity), whereas the other, 5'-GMGC-3'/3'-CGMG-5', displays cleavage activity approximately 6%.

D. The symmetrical palindromic site 5'-AMGT-3'/3'-TGMA-5' is a better substrate than those with three methylated cytosines, with two of these cytosines being external. We consider this site to be a good substrate of GlaI.

Results presented in Table 2 and Fig. 5 are further development of the enzyme GlaI characterization we began earlier [3,9]. In this study we have made some corrections in cleavage efficiency estimation for intermediate and low activity methylated substrates which have the general structure 5'-GCGC-3'/3'-CGCG-5'. We have also tested all possible 4-nucleotide DNA substrates. In the future we are planning to study in detail the influence of nucleotides which surround this site on the enzyme GlaI activity.

## Conclusion

Thus, the list of good substrates for GlaI includes a fully methylated site 5-CGCG-3'/3'-GCGC-5' (a canonical site, 100% cleavage activity), sites with three cytosines of a general structure 5'-PuMGM-3'/3'PyGMG-5' (27–61%) and one recognition sequence with two methylated cytosines 5'-AMGT-3'/3'-TGMA-5' (26%). GlaI intermediate substrates display activity which is 10–20% of the canonical one, and include sites with three methylated cytosines of a general structure 5'-GMPuM-3'/3'-MGPyG-5' as well as a site with two methylcytosines 5'-GMGT-3'/3'-CGMA-5'. The site 5'-GMGC-3'/3'-CGMG-5' displays about 6% activity and may be considered a low activity substrate. Although in some cases one strand of the intermediate substrates is cleaved with more than 20% activity, the total activity in duplex cleavage is less than 20% due to low cleavage activity of the second strand.

The role of GlaI, and similar IIM type enzymes, in a bacterial cell remains unclear. One possible function of these enzymes is protection against the invasion by foreign methylated phage DNA [2,3]. It is well known that in some phage DNAs there is a 5-methylcytosine in the nucleotide sequence 5'-GMGC-3'. This site is a substrate for methyl-directed DNA endonuclease GlaI [6]. However, according to the data of the current study, GlaI displays minimal activity in cleavage of recognition sequence 5'-GMGC-3'/3'-CGMG-5' and perhaps, this methyl-directed DNA endonuclease has some other function(s) in a bacterial cell.

GlaI may find a practical application in determination of DNA methylation status of eukaryotic and human genomes. Mammalian genomes contain the methylated dinucleotide 5'-CG-3' and study of this CG methylation in chromosomal DNA is in progress in many scientific institutions [10].

A present work has shown that GlaI recognizes a whole set of short 5mC-methylated nucleotide sequences. The list of good GlaI substrates includes recognition sequences with one methylated CG dinucleotide 5'-AMGT-3'/3'-TGMA-5'. Recognition sequences of other good substrates (5'-GMGM-3'/3'-MGMG-5' with four 5-methylcytosines and 5'-PuMGM-3'/3'PyGMG-5' with three 5-methylcytosines) include at least one methylated CG dinucleotide and may overlap with another 5mCG dinucleotide forming DNA sequence 5'-MGMG-3'. A general structure of such GlaI and two methylated CG dinucleotides overlapping sites is 5'-(A/G)CGCG-3', where the underlined sequence is the GlaI recognition site. Most GlaI intermediate activity substrates include either one methylated CG dinucleotide (5'-GMGT-3'/3'-CGMA-5') or may overlap with tetranucleotide 5'-MGMG-3' as well. Finally, GlaI low activity substrate 5'-GMGC-3'/3-CGMG-5' also contains methylated CG dinucleotide.

So, GlaI cleaves all these methylated DNA sequences and this enzyme may be used for analyzing methylation of specific CpG sites [11,12].

PCR-amplification of the genomic region of the interest will be more sensitive after pretreatment of DNA sample with GlaI endonuclease. Only the initially non-methylated and thus non-hydrolyzed fragment of DNA will be amplified. A detailed description of GlaI application will be provided in our forthcoming papers.

## Authors' contributions

TNN purified the enzyme. GVT carried out the experiments on oligonucleotides cleavage, SKD coordinated the project and prepared the final manuscript. All authors have read and approved the final manuscript.
